# IV-Thrombolysis in Ischemic Stroke With Unknown Time of Onset—Safety and Outcomes in Posterior vs. Anterior Circulation Stroke

**DOI:** 10.3389/fneur.2021.692067

**Published:** 2021-08-27

**Authors:** Kosmas Macha, Philip Hoelter, Gabriela Siedler, Ruihao Wang, Michael Knott, Svenja Stoll, Tobias Engelhorn, Arnd Doerfler, Stefan Schwab, Iris Mühlen, Bernd Kallmünzer

**Affiliations:** ^1^Department of Neurology, University Hospital Erlangen, Friedrich-Alexander-University of Erlangen-Nuremberg (FAU), Erlangen, Germany; ^2^Department of Neuroradiology, University Hospital Erlangen, Friedrich-Alexander-University of Erlangen-Nuremberg (FAU), Erlangen, Germany

**Keywords:** wake-up stroke, extended time window, IV-thrombolysis, posterior circulation stroke, anterior circulation stroke

## Abstract

**Background:** rt-PA for ischemic stroke in the unknown or extended time window beyond the first 4. 5 h after symptom onset is safe and effective for certain patients after selection by multimodal neuroimaging. However, the evidence for this approach comes mainly from patients with anterior circulation stroke (ACS), while the data on posterior circulation stroke (PCS) are scarce.

**Methods:** Ischemic stroke patients treated with IV-thrombolysis in the unknown or extended time window between January 2011 and May 2019 were identified from an institutional registry. The patients were categorized into PCS or ACS based on clinico-radiological findings. We analyzed the hemorrhagic complications, clinical and imaging efficacy outcomes, and mortality rates by comparing the PCS and ACS patient groups. Adjusted outcome analyses were performed after propensity score matching for the relevant factors.

**Results:** Of the 182 patients included, 38 (20.9%) had PCS and 144 (79.1%) had ACS. Symptomatic acute large vessel occlusion (LVO) was present in 123 patients on admission [27 (22.0%) PCS and 96 (78.0%) ACS]. The score on the National Institutes of Health Stroke Scale (NIHSS), the time from last seen normal, and the door-to-needle times were similar in PCS and ACS. In patients with LVO, the NIHSS score was lower [8 (5–15) vs. 14 (9–18), *p* = 0.005], and infarction visible on follow-up imaging was less common [70.4 vs. 87.5%; aRD, −18.9% (−39.8 to −2.2%)] in the PCS patient group. There was a trend toward a lower risk for intracranial hemorrhage (ICH) following intravenous thrombolysis in PCS vs. ACS, without reaching a statistical significance [5.3 vs. 16.9%; aRD, −10.4% (−20.4 to 4.0%)]. The incidence of symptomatic ICH [according to the ECASS III criteria: 2.6 vs. 3.5%; aRD, −2.9% (−10.3 to 9.2%)], efficacy outcomes, and mortality rates were similar in PCS and ACS patients.

**Conclusions:** In this real-world clinical cohort, the safety and the efficacy of rt-PA for ischemic stroke in the unknown or extended time window did not show relevant differences between PCS and ACS, with a trend toward less hemorrhagic complications in PCS. The findings reconfirm the clinician in the usage of rt-PA beyond the first 4.5 h also in selected patients with PCS.

## Introduction

In up to 16% of acute ischemic strokes treated with IV-thrombolysis, the territories of the posterior circulation, including the vertebral, basilar, or posterior cerebral arteries, are affected (1–4). In the subgroup of patients with severe ischemic stroke, presenting with National Institutes of Health Stroke Scale (NIHSS) scores >25, the proportion of PCS is increasing up to 36% (5). IV-thrombolysis is the standard of care for acute ischemic stroke within 4.5 h from symptom onset irrespective of the vascular territory affected (6, 7). In addition, several studies could demonstrate the safety and the efficacy of IV-thrombolysis for selected patients in the unknown or extended time window beyond 4.5 h (8–10). Multimodal CT or MRI was used for patient selection in these studies, and some of these approaches were implemented in the latest international guidelines (6–10).

The proportion of PCS was low or not reported in most randomized rt-PA trials with treatment within 4.5 h from onset and with treatment in the unknown or extended time window (8, 9, 11–14). Therefore, the transfer of the results to patients with PCS might be inappropriate.

A meta-analysis including 10,313 patients (PCS 11.9%) demonstrated a lower risk for symptomatic rt-PA-associated intracranial hemorrhage and higher rates of good functional outcome at 3 months in posterior circulation than in anterior circulation stroke patients receiving treatment in the approved time window of 4.5 h from onset (15).

The main objective of our study was to investigate these differences in the risk of hemorrhagic complications between PCS and ACS patients in the unknown or extended time window. The secondary objectives were imaging and functional efficacy outcomes and mortality rates.

## Materials and Methods

### Study Population, Procedures, and Analyzed Parameters

**Figure 1:**
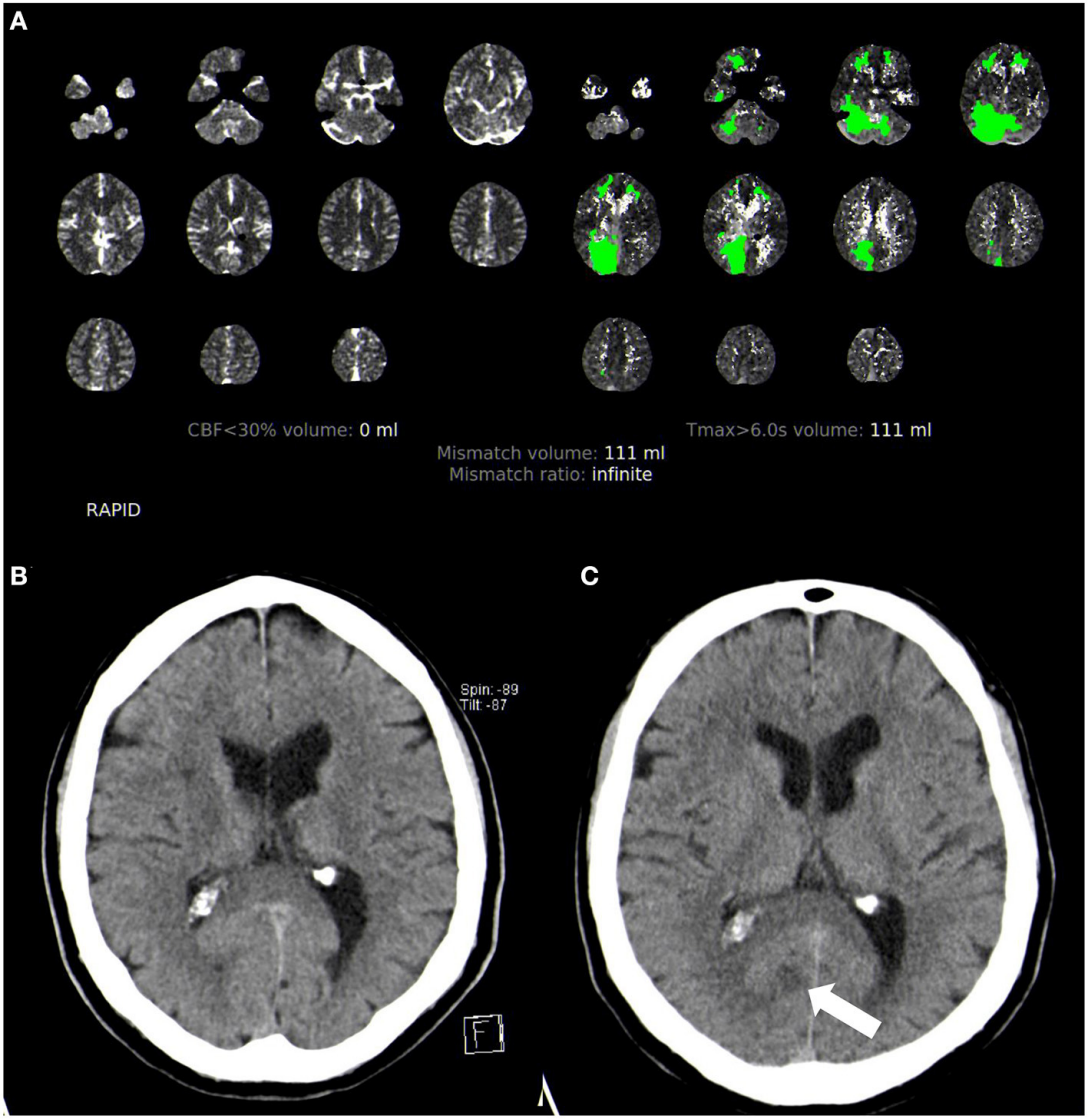
Acute multimodal CT and follow-up non-contrast CT imaging in posterior circulation stroke. **(A)** Perfusion CT analyzed with Rapid CTP. **(B)** Initial non-contrast CT showing no infarction. **(C)** Follow-up non-contrast CT with a visible small infarction in the posterior cerebral artery territory (arrow).

This retrospective cohort study is based on the data of consecutive acute ischemic stroke patients treated with IV-thrombolysis (rt-PA) in the unknown or extended time window >4.5 h in the period from January 2011 to May 2019 at our tertiary university stroke center. The patients were eligible for IV-thrombolysis using multimodal CT or MR imaging according to our institutional treatment algorithm for the management of ischemic stroke in the extended or unknown time window as described previously (16). Accordingly, IV-thrombolysis was performed after (1) the exclusion of intracranial hemorrhage and (2) the exclusion of major infarction on non-contrast CT or gradient echo and fluid-attenuated inversion recovery sequences using MRI and (3) evidence of salvageable tissue at risk on perfusion imaging (mismatch between hypoperfusion vs. infarcted core on perfusion CT or mismatch between perfusion-weighted and diffusion-weighted imaging using MRI; mismatch quotient >1.4 for both modalities) (Figure 1). Follow-up imaging was performed 24 h after IV-thrombolysis or earlier in the case of neurologic deterioration (NIHSS score increase of four points or more). The modality of follow-up imaging (CT or MRI) was to the discretion of the treating physician. We categorized all patients according to clinical and/or radiological findings as posterior circulation stroke or anterior circulation stroke patients (full patient cohort—clinico-radiological categorization); patients with simultaneous anterior and posterior circulation stroke were excluded. In addition, we conducted a subgroup analysis of patients with acute large vessel occlusion (LVO). Patients with occlusion of the internal carotid and middle or anterior cerebral artery were categorized as ACS patients, and patients with occlusion of the vertebral, basilar, or posterior cerebral artery were categorized as PCS patients. Patients with complete fetal posterior cerebral artery were excluded from the study.

We analyzed the clinical and imaging stroke characteristics, risk factors, procedure times for IV-thrombolysis and mechanical thrombectomy (if performed), clinical course including intracranial hemorrhagic complications, evidence of cerebral infarction on follow-up imaging, mortality rates, and 90-day outcome. Demographic, clinical, and radiologic data were collected during inpatient stay. The assessment of day 90 follow-up was conducted *via* telephone interview, outpatient visit, or medical reports by trained raters.

### Study Outcomes

The primary outcome was the incidence of hemorrhagic complication [any intracranial hemorrhage (ICH), symptomatic ICH according to different definitions—European Cooperative Acute Stroke Study (ECASS) III criteria (13), ECASS II criteria (11), and Safe Implementation of Thrombolysis in Stroke-Monitoring Study (SITS-MOST) criteria (12)] on follow-up imaging at 24 h after IV-thrombolysis.

The secondary outcomes were the rate of early major neurologic improvement (defined as a reduction of the NIHSS score by at least eight points or a score of 0 or 1 after 24 h), evidence of cerebral infarction on follow-up imaging after 24 h, mortality, and favorable functional outcome at day 90 using the modified Rankin Scale (mRS). A favorable outcome was defined as either a score on the mRS 0–2 or an improvement to the level prior to stroke onset, respectively.

### Statistics

Data were presented as absolute/relative numbers for categorical variables and median/interquartile range (IQR) for continuous variables. The significance of differences between patient groups (PCS and ACS) was calculated using the Mann–Whitney *U*-test, the χ^2^ test, and the Fisher exact test as appropriate. Statistical significance was set *a priori* at *p*-value < 0.05.

For the investigation of primary and secondary outcomes, adjusted analyses after propensity score matching (estimation algorithm: logistic regression, matching algorithm: nearest neighbor approach, caliper 0.2, match 1:3) were performed. The PCS and ACS patients were matched for age, female sex, arterial hypertension, diabetes mellitus, atrial fibrillation, NIHSS on admission, large vessel occlusion (in clinico-radiological cohort), and mechanical thrombectomy (Supplementary Figures 1, 2). We calculated absolute differences between PCS and ACS patients in percentage (absolute risk difference, aRD) with corresponding 95% confidence interval. Negative values indicate a decrease of measurement from ACS patients as reference.

Missing data of the 90-day follow-up were excluded from the outcome analyses. Statistical analyses were performed using IBM SPSS Statistics 21 software package (www.spss.com) and R 2.12.1 (www.r-project.org).

## Results

**Figure 2:**
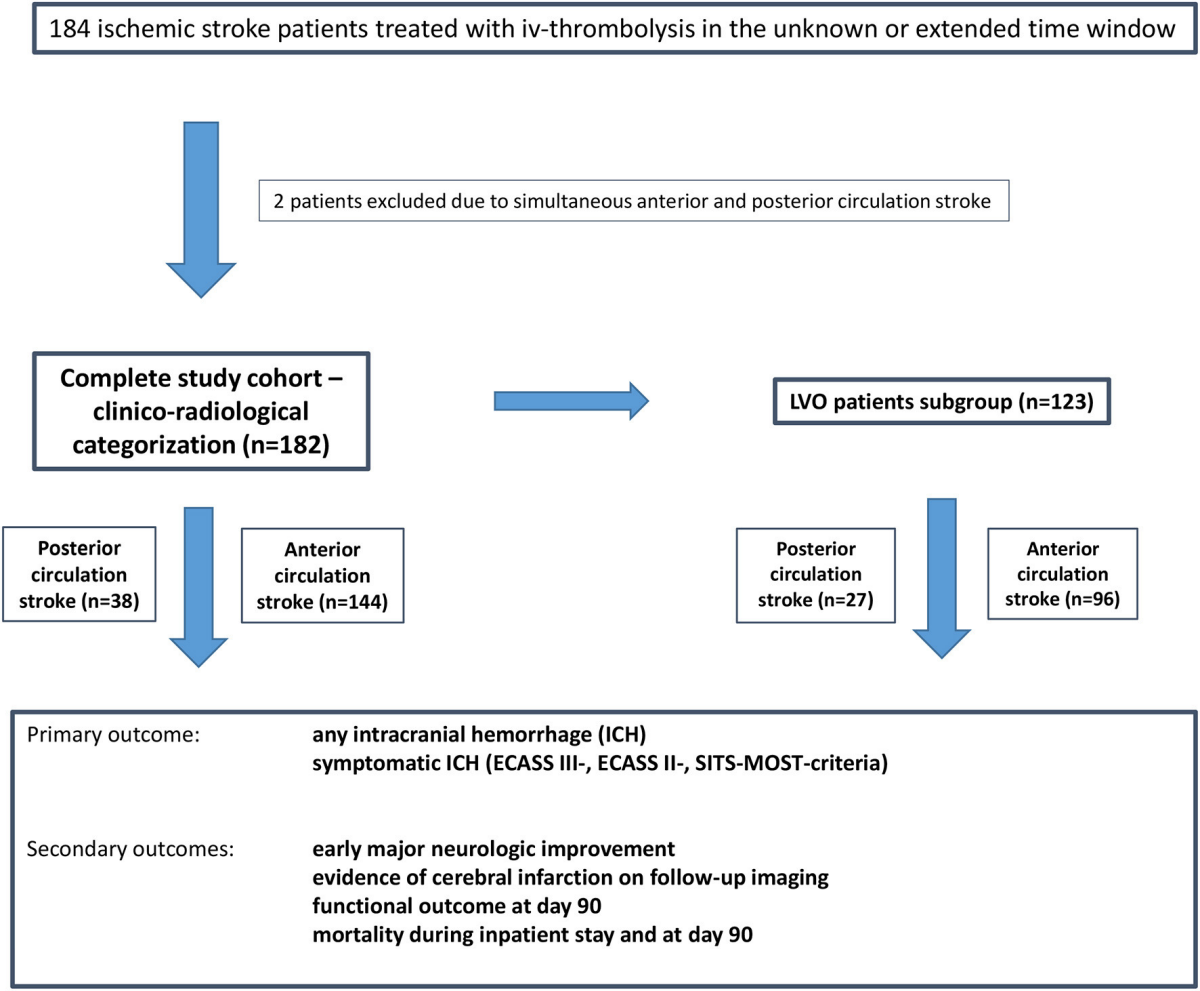
Flow chart of the study cohort.

One hundred eighty-four acute ischemic stroke patients treated with IV-thrombolysis in the unknown or extended time window were identified. Two patients were excluded due to simultaneous anterior and posterior circulation stroke, resulting in the final study cohort of 182 patients including 38 (20.9%) PCS and 144 (79.1%) ACS patients (Figure 2). Acute LVO was present in 123 patients (67.6%) and constituted the LVO patient subgroup.

### Full Patient Cohort—Clinico-Radiological Categorization

Patients with PCS were less commonly female (28.9 vs. 56.9%, *p* = 0.002) but similar regarding comorbidities and concurrent medication (Table 1). The proportion of patients in the confirmed time window >4.5 h was higher in the PCS group (23.7 vs. 9.0%, *p* = 0.023), and the time from last seen normal to treatment was similar in PCS and ACS patients [450 min (362–719) vs. 558 min (354–819), *p* = 0.148]. Differences in NIHSS scores on admission between PCS and ACS patients [8 (5–14) vs. 11 (6–17), *p* = 0.068] did not reach statistical significance. Door-to-needle times were similar in PCS and ACS patients [56 min (42–91) vs. 59 min (41–76), *p* = 0.510]. Further clinical and imaging characteristics of stroke are shown in Table 2.

**Table 1:** Baseline characteristics in the clinico-radiological patient cohort.

	**Posterior circulation stroke (*n* = 38)**	**Anterior circulation stroke (*n* = 144)**	***p*-value**
Female sex, *n* (%)	11 (28.9)	82 (56.9)	0.002
Age in years, median (IQR)	77.5 (67.0–84.0)	80.0 (69.0–86.0)	0.268
Arterial hypertension, *n* (%)	32 (84.2)	124 (86.1)	0.796
Hyperlipidemia, *n* (%)	22 (57.9)	68 (47.2)	0.242
Diabetes mellitus, *n* (%)	12 (31.6)	37 (25.7)	0.467
History of stroke, *n* (%)	10 (26.3)	28 (19.4)	0.354
Atrial fibrillation, *n* (%)	12 (31.6)	64 (44.4)	0.153
Antiplatelets, *n* (%)	15 (39.5)	49 (34.0)	0.532
Oral anticoagulants, *n* (%)	1 (2.6)	15 (10.4)	0.199
Vitamin K antagonists, *n* (%)	1 (2.6)	6 (4.2)	1.000
Direct oral anticoagulants, *n* (%)	0 (0.0)	9 (6.3)	0.207
Pre-mRS score, median (IQR)	1 (0–3)	1 (0–3)	0.859

*pre-mRS, pre-stroke modified Rankin scale*.

**Table 2:** Clinical and imaging characteristics of stroke in the clinico-radiological patient cohort.

	**Posterior circulation stroke (*n* = 38)**	**Anterior circulation stroke (*n* = 144)**	***p*-value**
Last seen normal to door—min, median (IQR)	382 (283–681)	479 (301–746)	0.098
Symptom recognition to door—min, median (IQR)	74 (64–128)	74 (51–140)	0.511
Confirmed extended time window, *n* (%)	9 (23.7)	13 (9.0)	0.023
Symptom onset to door in patients in the extended time window—min, median (IQR)	284 (245–382)	309 (265–495)	0.262
Patients transferred from external hospital for treatment, *n* (%)	2 (5.3)	9 (6.3)	1.000
Multimodal CT imaging for thrombolysis, *n* (%)	23 (60.5)	76 (52.8)	0.394
NIHSS score on admission, median (IQR)	8 (5–14)	11 (6–17)	0.068
NIHSS score >25 on admission, *n* (%)	3 (7.9)	3 (2.1)	0.106
Intubated on admission, *n* (%)	2 (5.3)	5 (3.5)	0.637
Wake-up stroke, *n* (%)	23 (60.5)	101 (70.1)	0.258
Infra- and supratentorial stroke, *n* (%)	13 (34.2)		
Large vessel occlusion, *n* (%)	27 (71.1)	96 (66.7)	0.607
Volume of irreversibly injured ischemic core tissue at initial imaging^a,b^–ml, median (IQR)	0.0 (0.0–0.0)	0.0 (0.0–10.5)	0.001
Perfusion lesion volume at initial imaging^b,c^–ml, median (IQR)	36.0 (11.0–129.0)	45.0 (10.0–109.5)	0.916
Mechanical thrombectomy, *n* (%)	13 (34.2)	49 (34.0)	0.983
Additional intra-arterial thrombolysis, *n* (%)	3 (7.9)	3 (2.1)	0.106
Door to needle—min, median (IQR)	56 (42–91)	59 (41–76)	0.510
Last seen normal to needle—min, median (IQR)	450 (362–719)	558 (354–819)	0.148
Symptom recognition to needle—min, median (IQR)	143 (112–177)	145 (110–211)	0.675
Follow-up imaging MRI, *n* (%)	2 (5.3)	11 (7.7)	1.000

*NIHSS, NIH Stroke Scale; rtPa, recombinant tissue plasminogen activator. Two anterior circulation stroke patients died prior to follow-up imaging*.

a *The volume of irreversibly injured ischemic core tissue was calculated with the use of a threshold for relative cerebral blood flow of less than 30% of that in normal brain tissue or with the use of diffusion-weighted MRI (apparent diffusion coefficient)*.

b *The volumetric assessment of perfusion imaging was available in 27 posterior circulation stroke and 101 anterior circulation stroke patients*.

c *To define the critically hypoperfused tissue, the perfusion lesion volume was calculated as the volume of tissue in which there had been a delayed arrival of an injected tracer agent exceeding 6 s*.

**Figure 3:**
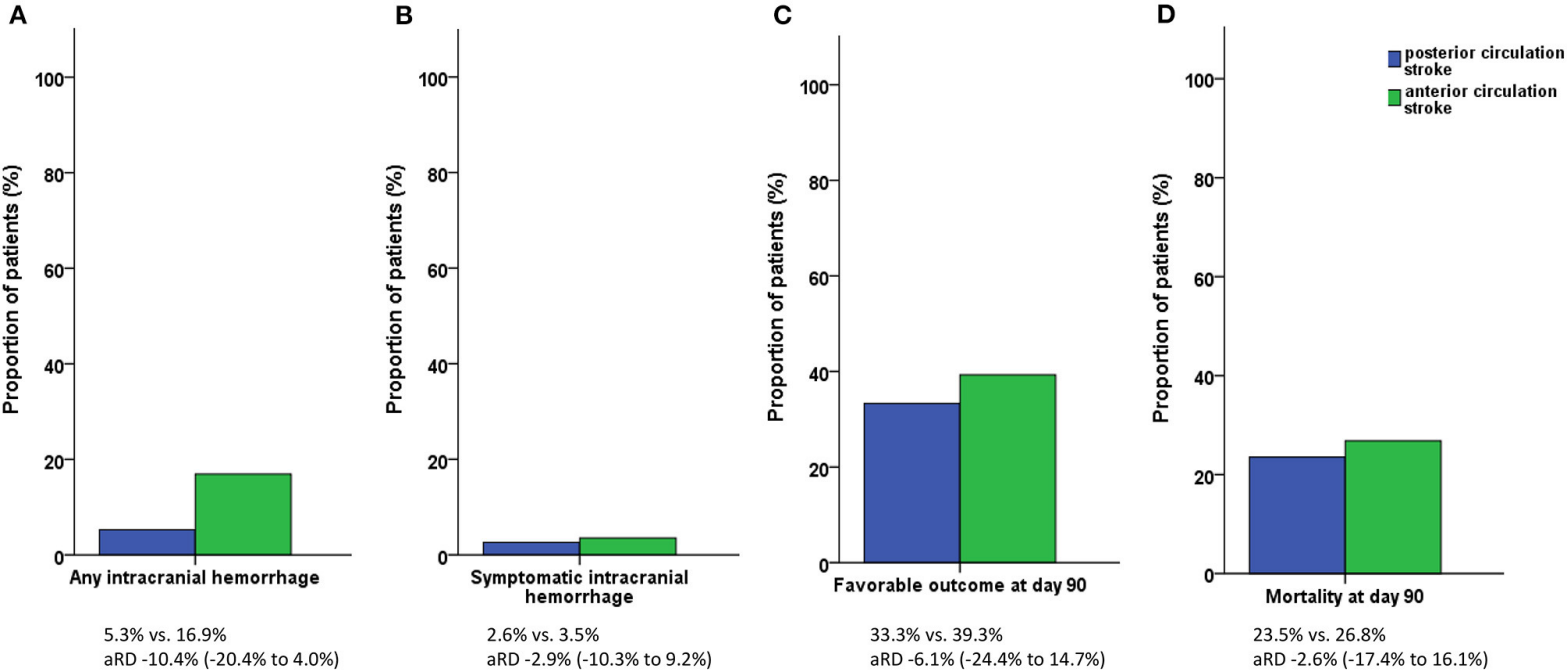
Study outcomes. **(A)** Incidence of any intracranial hemorrhage. **(B)** Incidence of symptomatic intracranial hemorrhage. **(C)** Functional outcome at day 90. **(D)** Mortality at day 90. Symptomatic intracranial hemorrhage according to the ECASS III criteria. Favorable outcome defined as pre-modified Rankin Scale (mRS) or mRS score 0–2.

There was a statistical trend for a lower incidence of any intracranial hemorrhage post IV-thrombolysis in PCS than in ACS patients (5.3 vs. 16.9%, *p* = 0.075). The proportion of patients with symptomatic ICH following different definitions (ECASS III criteria: 2.6 vs. 3.5%, *p* = 1.000) and fatal ICH (0.0 vs. 1.4%, *p* = 1.000) was similar in PCS and ACS patients. There was no significant difference in the rate of infarction visible on follow-up imaging (65.8 vs. 77.5%, *p* = 0.140), early neurologic improvement (15.8 vs. 18.1%, *p* = 1.000), or functional outcome at day 90 (pre-mRS or mRS score 0–2: 33.3 vs. 39.3%, *p* = 0.532). The mortality rates during inpatient stay and at day 90 were similar in PCS and ACS patients (13.2 vs. 11.8%, *p* = 0.784 and 23.5 vs. 26.8%, *p* = 0.827) (Figure 3). Two patients in the ACS group died prior to follow-up imaging (the suspected cause of death was lung embolism and circulatory failure, respectively).

The analyses after propensity score matching included 124 patients (PCS, *n* = 35; ACS, *n* = 89) and did not lead to statistical differences in the incidence of any ICH [aRD −10.4% (−20.4 to 4.0%)], symptomatic ICH, evidence of infarction on follow-up imaging, early neurologic improvement, functional outcome, or mortality (Table 3).

**Table 3:** Outcomes in the clinico-radiological patient cohort.

	**Posterior circulation stroke (*n* = 38)**	**Anterior circulation stroke (*n* = 144)**	***p*-value**	**aRD^**a**^**	**95% CI**
Any ICH, *n* (%)	2 (5.3)	24 (16.9)	0.075	−10.4%	−20.4 to 4.0%
Symptomatic ICH according to ECASS III criteria^b^, *n* (%)	1 (2.6)	5 (3.5)	1.000	−2.9%	−10.3 to 9.2%
Symptomatic ICH according to ECASS II criteria^c^, *n* (%)	1 (2.6)	6 (4.2)	1.000	−2.9%	−10.3 to 9.2%
Symptomatic ICH according to SITS-MOST criteria^d^, *n* (%)	0 (0.0)	3 (2.1)	1.000	−3.4%	−9.7 to 6.7%
Fatal ICH, *n* (%)	0 (0.0)	2 (1.4)	1.000	−2.3%	−8.0 to 7.7%
ICH location remote^e^, *n* (%)	2 (5.3)	7 (4.9)	1.000	–	–
Infarction on follow-up imaging, *n* (%)	25 (65.8)	110 (77.5)	0.140	−9.6%	−27.7 to 6.6%
Early major neurologic improvement^f^, *n* (%)	6 (15.8)	26 (18.1)	1.000	1.4%	−11.3 to 18.1%
Mortality during inpatient stay, *n* (%)	5 (13.2)	17 (11.8)	0.784	3.6%	−6.6 to 18.6%
Favorable outcome at day 90 (pre-mRS or mRS score: 0–2)^g^, *n* (%)	11 (33.3)	46 (39.3)	0.532	−6.1%	−24.4 to 14.7%
90-day mortality^h^, *n* (%)	8 (23.5)	33 (26.8)	0.827	−2.6%	−17.4 to 16.1%

*aRD, adjusted risk difference; ICH, intracranial hemorrhage; mRS, modified Rankin scale; pre-mRS, pre-stroke modified Rankin scale. Two anterior circulation stroke patients died prior to follow-up imaging*.

a *Adjusted for age, female sex, arterial hypertension, diabetes mellitus, atrial fibrillation, NIHSS on admission, large vessel occlusion, and mechanical thrombectomy using propensity score matching; posterior circulation stroke (PCS), n = 35; anterior circulation stroke (ACS), n = 89*.

b *Any intracranial hemorrhage identified as a predominant cause of neurologic deterioration as indicated by a NIHSS score increase of four or more points from baseline or the lowest score or any hemorrhage leading to death*.

c *Any intracranial hemorrhage with neurologic deterioration as indicated by a NIHSS score increase of four or more points from baseline or the lowest score or any hemorrhage leading to death*.

d *Local or remote parenchymal hematoma type 2 and neurologic deterioration as indicated by a NIHSS score increase of four or more points from baseline or the lowest score or hemorrhage leading to death*.

e *Two patients with remote ICH had an additional peri-ischemic hemorrhage (one PCS patient and one ACS patient)*.

f *Early major neurologic improvement defined as a reduction of NIHSS score of at least eight points or a score of 0 or 1 at 24 h*.

g *The 90-day mRS was available in 150 patients (82.4%; 33 posterior circulation stroke patients and 117 anterior circulation stroke patients)*.

h *Vital status at day 90 was available in 157 patients (86.3%; 34 posterior circulation stroke patients and 123 anterior circulation stroke patients)*.

### LVO Patient Subgroup

Among a total of 123 patients with acute large vessel occlusion, 27 (22.0%) were PCS patients and 96 (78.0%) were ACS patients. The distribution of occlusion sites in PCS and ACS patients is given in Table 4. The PCS patients were less commonly female (29.6 vs. 59.4%, *p* = 0.008). There were no significant differences in comorbidities and concurrent medication (Table 5). The proportion of patients in the confirmed extended time window beyond 4.5 h was higher in PCS patients (22.2 vs. 6.3%, *p* = 0.023), and the time from symptom recognition to admission was shorter in ACS patients [68 min (50–123) vs. 89 min (67–153), *p* = 0.049]. The NIHSS scores on admission were higher in ACS patients [14 (9–18) vs. 8 (5–15), *p* = 0.005]. The time from last seen normal to the start of IV-thrombolysis did not differ significantly between the PCS and ACS patients [457 min (368–745) vs. 613 (385–843), *p* = 0.141]; mechanical thrombectomy was equally performed in the PCS and ACS patient groups (48.1 vs. 51.0%, *p* = 0.790). The door-to-needle times [55 min (42–91) vs. 61 min (42–78), *p* = 0.886] and the door-to-groin times [97 min (78–121) vs. 94 min (77–111), *p* = 0.621] were similar in PCS and ACS patients. Further clinical and imaging characteristics of stroke are shown in Table 6. The incidence of any ICH (7.4 vs. 18.9%, *p* = 0.239), symptomatic ICH (ECASS III criteria: 3.7 vs. 2.1%, *p* = 0.531), and fatal ICH (0.0 vs. 1.0%, *p* = 1.000) was similar in PCS and ACS patients. Infarction on follow-up imaging was significantly more often evident in ACS patients than in PCS patients (87.5 vs. 70.4%, *p* = 0.034). Early neurologic improvement (18.5 vs. 16.7%, *p* = 0.779), functional outcome at day 90 (pre-mRS or mRS score 0–2: 21.7 vs. 28.9%, *p* = 0.603), and mortality rates (inpatient stay: 18.5 vs. 14.6%, *p* = 0.563; day 90: 29.2 vs. 30.7%, *p* = 1.000) were similar in PCS and ACS patients. One ACS patient died prior to follow-up imaging (the suspected cause of death was lung embolism).

**Table 4:** Distribution of large vessel occlusion (LVO) in the LVO patient cohort.

**Occlusion site**	**PCS (*n* = 27)**	**ACS (*n* = 96)**
Internal carotid artery (ICA)	–	14 (14.6%)^b^
Carotid-T	–	7 (7.3%)^b^
M1 segment of middle cerebral artery (MCA)	–	48 (50.0%)^b^
M2/M3 segment of MCA	–	28 (29.2%)^b^
Anterior cerebral artery (ACA)	–	2 (2.1%)^b^
Vertebral artery (VA)	8 (29.6%)^a^	–
Basilar artery (BA)	11 (40.7%)^a^	–
Posterior cerebral artery (PCA)	12 (44.4%)^a^	—

a *The PCA group included one patient with combined VA/PCA occlusion, two patients with combined BA/PCA occlusion, and one patient with combined VA/BA occlusion*.

b *The ACS group included two patients with combined ICA/M1 occlusion and one patient with combined M1/M2 occlusion*.

**Table 5:** Baseline characteristics in the large vessel occlusion patient cohort.

	**Posterior circulation stroke (*n* = 27)**	**Anterior circulation stroke (*n* = 96)**	***p*-value**
Female sex, *n* (%)	8 (29.6)	57 (59.4)	0.008
Age in years, median (IQR)	77.0 (69.0–82.0)	80.0 (71.0–87.0)	0.179
Arterial hypertension, *n* (%)	23 (85.2)	85 (88.5)	0.739
Hyperlipidemia, *n* (%)	17 (63.0)	43 (44.8)	0.095
Diabetes mellitus, *n* (%)	12 (44.4)	26 (27.1)	0.085
History of stroke, *n* (%)	7 (25.9)	18 (18.8)	0.425
Atrial fibrillation, *n* (%)	9 (33.3)	52 (54.2)	0.081
Antiplatelets, *n* (%)	10 (37.0)	31 (32.3)	0.644
Oral anticoagulants, *n* (%)	1 (3.7)	12 (12.5)	0.294
Vitamin K antagonists, *n* (%)	1 (3.7)	5 (5.2)	1.000
Direct oral anticoagulants, *n* (%)	0 (0.0)	7 (7.3)	0.346
Pre-mRS score, median (IQR)	1 (0–2)	1 (0–3)	0.547

*pre-mRS, pre-stroke modified Rankin scale*.

**Table 6:** Clinical and imaging characteristics of stroke in the large vessel occlusion patient cohort.

	**Posterior circulation stroke (*n* = 27)**	**Anterior circulation stroke (*n* = 96)**	***p*-value**
Last seen normal to door—min, median (IQR)	396 (318–693)	533 (320–804)	0.127
Symptom recognition to door—min, median (IQR)	89 (67–153)	68 (50–123)	0.049
Confirmed extended time window, *n* (%)	6 (22.2)	6 (6.3)	0.023
Symptom onset to door in patients in the extended time window—min, median (IQR)	349 (256–491)	398 (299–636)	0.485
Patients transferred from external hospital for treatment, *n* (%)	1 (3.7)	7 (7.3)	0.684
Multimodal CT imaging for thrombolysis, *n* (%)	17 (63.0)	47 (49.0)	0.198
NIHSS score on admission, median (IQR)	8 (5–15)	14 (9–18)	0.005
NIHSS score >25 on admission, *n* (%)	3 (11.1)	3 (3.1)	0.119
Intubated on admission, *n* (%)	2 (7.4)	5 (5.2)	0.648
Wake-up stroke, *n* (%)	16 (59.3)	72 (75.0)	0.109
Infra- and supratentorial stroke, *n* (%)	11 (40.7)		
Volume of irreversibly injured ischemic core tissue at initial imaging^a,b^–ml, median (IQR)	0.0 (0.0–0.0)	0.0 (0.0–18.0)	0.001
Perfusion lesion volume at initial imaging^b,c^–ml, median (IQR)	46.0 (19.0–125.0)	87.0 (38.0–153.0)	0.132
Mechanical thrombectomy, *n* (%)	13 (48.1)	49 (51.0)	0.790
Additional intra-arterial thrombolysis, *n* (%)	3 (11.1)	3 (3.1)	0.119
Recanalization TICI 2b/3 in EVT patients	12/13 (92.3)	44/49 (89.8)	1.000
Recanalization after IVT prior to EVT	1/13 (2.0)	2/49 (15.4)	0.109
Door to needle—min, median (IQR)	55 (42–91)	61 (42–78)	0.886
Last seen normal to needle—min, median (IQR)	457 (368–745)	613 (385–843)	0.141
Symptom recognition to needle—min, median (IQR)	145 (115–237)	133 (107–194)	0.264
Door to groin^d^–min, median (IQR)	97 (78–121)	94 (77–111)	0.621
Last seen normal to recanalization^e^–min, median (IQR)	575 (521–958)	805 (525–970)	0.427
Symptom recognition to recanalization^e^–min, median (IQR)	260 (205–360)	265 (195–335)	0.929
Follow-up imaging MRI, *n* (%)	1 (3.7)	4 (4.2)	1.000

*NIHSS, NIH Stroke Scale; rtPa, recombinant tissue plasminogen activator. One anterior circulation stroke patient died prior to follow-up imaging*.

a *The volume of irreversibly injured ischemic core tissue was calculated with the use of a threshold for relative cerebral blood flow of less than 30% of that in normal brain tissue or with the use of diffusion-weighted MRI (apparent diffusion coefficient)*.

b *The volumetric assessment of perfusion imaging was available in 20 posterior circulation stroke and 65 anterior circulation stroke patients*.

c *To define the critically hypoperfused tissue, perfusion lesion volume was calculated as the volume of tissue in which there had been a delayed arrival of an injected tracer agent exceeding 6 s*.

d *The time of groin puncture was available in 11 posterior circulation stroke (PCS) and 44 anterior circulation stroke (ACS) patients treated with additional mechanical thrombectomy*.

e *The time of recanalization was available in eight PCS patients and 39 ACS patients treated with additional mechanical thrombectomy*.

In the analyses after propensity score matching (the total included 80 patients: PCS, *n* = 23; ACS, *n* = 57), the lower rate of infarction on follow-up imaging in PCS patients remained statistically significant [aRD −18.9% (−39.5 to −2.2%)]. No significant differences were detected regarding any ICH [aRD −7.4% (−20.7 to 12.2%)], symptomatic ICH, fatal ICH, and early or late neurologic/functional outcome or mortality rates (Table 7).

**Table 7:** Outcomes in the large vessel occlusion patient cohort.

	**Posterior circulation stroke (*n* = 27)**	**Anterior circulation stroke (*n* = 96)**	***p*-value**	**aRD^**a**^**	**95% CI**
Any ICH, *n* (%)	2 (7.4)	18 (18.9)	0.239	−7.4%	−20.7 to 12.2%
Symptomatic ICH according to ECASS III criteria^b^, *n* (%)	1 (3.7)	2 (2.1)	0.531	0.8%	−8.5 to 17.6%
Symptomatic ICH according to ECASS II criteria^c^, *n* (%)	1 (3.7)	3 (3.2)	1.000	0.8%	−8.5 to 17.6%
Symptomatic ICH according to SITS-MOST criteria^d^, *n* (%)	0 (0.0)	2 (2.1)	1.000	−3.6%	−12.1 to 11.0%
Fatal ICH, *n* (%)	0 (0.0)	1 (1.0)	1.000	−1.8%	−9.4 to 12.6%
ICH location remote^e^, *n* (%)	2 (7.4)	5 (5.3)	0.650	–	–
Infarction on follow-up imaging, *n* (%)	19 (70.4)	84 (87.5)	0.034	−18.9%	−39.8 to −2.2%
Early major neurologic improvement^f^, *n* (%)	5 (18.5)	16 (16.7)	0.779	5.9%	−10.8 to 27.4%
Mortality during inpatient stay, *n* (%)	5 (18.5)	14 (14.6)	0.563	5.1%	−10.0 to 25.8%
Favorable outcome at day 90 (pre-mRS or mRS score 0–2)^g^, *n* (%)	5 (21.7)	24 (28.9)	0.603	−15.5%	−34.0 to 9.7%
90-day mortality^h^, *n* (%)	7 (29.2)	27 (30.7)	1.000	−1.9%	−21.1 to 22.2%

*aRD, adjusted risk difference; ICH, intracranial hemorrhage; mRS, modified Rankin scale; pre-mRS, pre-stroke modified Rankin scale. One anterior circulation stroke patient died prior to follow-up imaging*.

a *Adjusted for age, female sex, arterial hypertension, diabetes mellitus, atrial fibrillation, NIHSS on admission, and mechanical thrombectomy using propensity score matching; posterior circulation stroke (PCS) n = 23, anterior circulation stroke (ACS) n = 57*.

b *Any intracranial hemorrhage identified as a predominant cause of neurologic deterioration as indicated by a NIHSS score increase of four or more points from baseline or the lowest score or any hemorrhage leading to death*.

c *Any intracranial hemorrhage with neurologic deterioration as indicated by a NIHSS score increase of four or more points from baseline or the lowest score or any hemorrhage leading to death*.

d *Local or remote parenchymal hematoma type 2 and neurologic deterioration as indicated by a NIHSS score increase of four or more points from baseline or the lowest score or hemorrhage leading to death*.

e *Two patients with remote ICH had an additional peri-ischemic hemorrhage (one PCS patient and one ACS patient)*.

f *Early major neurologic improvement defined as a reduction of NIHSS score of at least eight points or a score of 0 or 1 at 24 h*.

g *The 90-day mRS was available in 106 patients (86.2%; 23 posterior circulation stroke patients and 83 anterior circulation stroke patients)*.

h *Vital status at day 90 was available in 112 patients (91.1%; 24 posterior circulation stroke patients and 88 anterior circulation stroke patients)*.

## Discussion

The major findings of our real-world clinical cohort study investigating the treatment of acute ischemic stroke with IV-thrombolysis in the unknown or extended time window are as follows: (1) the rates of symptomatic ICH were low, without a significant difference between PCS and ACS patients, (2) there was a trend toward more hemorrhages among ACS patients than in PCS patients, and (3) the functional outcome and mortality rates at day 90 did not differ.

The rates of symptomatic ICH in our study were similar to the ones reported in the literature, without a difference between PCS and ACS patients (2, 3). Controversially, a large meta-analysis including 10,313 patients (1,224 PCS) reported the risk for sICH in PCS as half of that in ACS (15). However, while the rate of symptomatic hemorrhages did not differ in our cohort, the incidence of any ICH (including asymptomatic ones) seemed slightly higher in ACS, without reaching statistical significance. The higher volumes of ischemic core on initial multimodal imaging may have contributed to the higher risk for hemorrhagic complications in ACS patients, as infarct size is an established risk factor for hemorrhagic transformation (17, 18). These differences in ischemic core on initial imaging might be responsible for the higher rates of cerebral infarction visible on follow-up imaging in ACS patients of our LVO cohort. In addition, cerebral infarction in the posterior circulation including the brainstem might be more difficult to detect compared to anterior circulation stroke infarction on follow-up imaging (19). The rates of MRI for follow-up imaging, improving the detection of infarction especially in the posterior cranial fossa, were low and did not differ between PCS and ACS patients.

The differences in infarction on follow-up imaging did not translate into a functional outcome in PCS and ACS patients in our LVO patient cohort. The absence of correlation between the volume of infarcted tissue and the functional outcome was reported earlier for LVO patients receiving recanalization therapy (20).

Several studies demonstrated similar functional outcomes in PCS and ACS patients (1–3, 15, 21). Consistently, we did not find differences in outcome between patient groups in the analysis of the full patient cohort.

The mortality rates in PCS and ACS are reported inconsistently in the literature (2, 3, 15). This heterogeneity may be attributed to differences in the proportion of severe stroke patients between study cohorts in ACS and PCS patients. In our study, the proportion of severe stroke patients presenting with NIHSS score >25 on admission seemed higher in PCS patients, without reaching statistical significance. Nevertheless, we found no difference in mortality rates at day 90 between PCS and ACS patients in our study.

The proportion of patients with PCS in our study was slightly higher than the one reported in previous cohorts, including patients within 4.5 h from symptom onset (1–4, 15). Considering the poor prognosis of severe posterior circulation stroke without recanalizing therapies, the higher proportion of PCS in our cohort might refer to the aggressive treatment also beyond the first 4.5 h after onset (5, 22). Furthermore, a higher proportion of patients treated in the confirmed extended time window >4.5 h in PCS might have contributed to this finding and leads to the higher proportion of PCS patients in our cohort.

The higher proportion of female sex in ACS patients in our study was consistent to previous findings, demonstrating female sex as a known risk factor of ACS (15, 23). The PCS patients presented with lower median NIHSS scores on admission statistically significant in LVO patients confirming a known underrepresentation of PCS symptoms in the NIHSS (15, 21, 24).

As the symptoms of PCS can be clinically less noticeable, this may have led to longer times from symptom recognition to door in LVO patients of our study. Less noticeable symptoms may have led to longer door-to-needle times reported for PCS patients compared to ACS previously (25). In our study, the procedure times did not differ significantly between PCS and ACS patients despite the lower NIHSS scores on admission in PCS patients. Similar procedure times in ACS and PCS patients might be another evidence for an aggressive treatment of PCS patients in our cohort. Overall, the times from last seen normal to treatment were similar in PCS and ACS patients in our study.

Our study has relevant limitations, mainly its small patient number and monocentric design. The limited sample size might be causal for missing statistical significance in the difference of any ICH in our study. Furthermore, the classification as posterior or anterior circulation stroke based on clinical symptoms can be challenging in some patients presenting without LVO and with no infarction visible on follow-up imaging. This may have led to wrongly classifying some patients in the clinico-radiological cohort. However, no clear contradictory results between our clinico-radiological and LVO patient cohorts regarding primary and secondary outcomes were detected.

To conclude the main result of our study, the rates of hemorrhagic complications were low in PCS patients treated with IV-thrombolysis in the unknown or extended time window. This will reaffirm the clinician in the use of rt-PA for selected PCS patients beyond the established 4.5-h time window.

## Data Availability Statement

Anonymized data will be shared on request to any qualified investigator.

## Ethics Statement

The studies involving human participants were reviewed and approved by the Ethics Board, Medical Faculty, University of Erlangen-Nuremberg. Written informed consent for participation was not required for this study in accordance with the national legislation and the institutional requirements.

## Author Contributions

KM contributed to the design and conceptualization of the study, acquisition and analysis of data, and drafting of the manuscript for intellectual content. PH, GS, RW, MK, and SS contributed to the acquisition of data and revision of the manuscript for intellectual content. TE, AD, and SS contributed to the design and conceptualization of the study and revision of the manuscript for intellectual content. IM contributed to the design and conceptualization of the study, analysis of data, and revision of the manuscript for intellectual content. BK contributed to the design and conceptualization of the study, analysis of data, and drafting of the manuscript for intellectual content. All authors contributed to the article and approved the submitted version.

## Conflict of Interest

The authors declare that the research was conducted in the absence of any commercial or financial relationships that could be construed as a potential conflict of interest.

## Publisher's Note

All claims expressed in this article are solely those of the authors and do not necessarily represent those of their affiliated organizations, or those of the publisher, the editors and the reviewers. Any product that may be evaluated in this article, or claim that may be made by its manufacturer, is not guaranteed or endorsed by the publisher.

## Supplementary Material

Supplemental Figure 1 Standardized differences in variables included in the propensity score before and after matching in the clinico-radiologic patient cohort.

Supplemental Figure 2 Standardized differences in variables included in the propensity score before and after matching in the large vessel occlusion patient cohort.

## References

[1] Sarikaya H Arnold M Engelter ST Lyrer PA Mattle HP Georgiadis D. Outcomes of intravenous thrombolysis in posterior versus anterior circulation stroke. Stroke. (2011) 42:2498–502. 10.1161/STROKEAHA.110.607614 21778443

[2] Breuer L Huttner HB Jentsch K Blinzler C Winder K Engelhorn T. Intravenous thrombolysis in posterior cerebral artery infarctions. Cerebrovasc Dis. (2011) 31:448–54. 10.1159/000323253 21346350

[3] Förster A Gass A Kern R Griebe M Hennerici MG Szabo K. Thrombolysis in posterior circulation stroke: stroke subtypes and patterns, complications and outcome. Cerebrovasc Dis. (2011) 32:349–53. 10.1159/000330346 21921598

[4] Sung S-F Chen C-H Chen Y-W Tseng M-C Shen H-C Lin H-J. Predicting symptomatic intracerebral hemorrhage after intravenous thrombolysis: stroke territory as a potential pitfall. J Neurol Sci. (2013) 335:96–100. 10.1016/j.jns.2013.08.036 24054716

[5] Mazya MV Lees KR Collas D Rand V-M Mikulik R Toni D. IV thrombolysis in very severe and severe ischemic stroke: results from the SITS-ISTR registry. Neurology. (2015) 85:2098–106. 10.1212/WNL.0000000000002199 26546630 PMC4691682

[6] Powers WJ Rabinstein AA Ackerson T Adeoye OM Bambakidis NC Becker K. Guidelines for the early management of patients with acute ischemic stroke: 2019 update to the 2018 guidelines for the early management of acute Ischemic stroke: a guideline for healthcare professionals from the American Heart Association/American Stroke Association. Stroke. (2019) 50:e344–418. 10.1161/STROKEAHA.119.026917 31662037

[7] Berge E Whiteley W Audebert H Marchis GMD Fonseca AC Padiglioni C. European Stroke Organisation (ESO) guidelines on intravenous thrombolysis for acute ischaemic stroke. Eur Stroke J. 2021:2396987321989865. 10.1177/2396987321989865 33817340 PMC7995316

[8] Thomalla G Simonsen CZ Boutitie F Andersen G Berthezene Y Cheng B. MRI-guided thrombolysis for stroke with unknown time of onset. N Engl J Med. (2018) 379:611–22. 10.1056/NEJMoa1804355 29766770

[9] Ma H Campbell BCV Parsons MW Churilov L Levi CR Hsu C. Thrombolysis guided by perfusion imaging up to 9 hours after onset of stroke. N Engl J Med. (2019) 380:1795–803. 10.1056/NEJMoa1813046 31067369

[10] Campbell BCV Ma H Ringleb PA Parsons MW Churilov L Bendszus M. Extending thrombolysis to 4.5–9 h and wake-up stroke using perfusion imaging: a systematic review and meta-analysis of individual patient data. Lancet. (2019) 394:139–47. 10.1016/S0140-6736(19)31053-0 31128925

[11] Hacke W Kaste M Fieschi C von Kummer R Davalos A Meier D. Randomised double-blind placebo-controlled trial of thrombolytic therapy with intravenous alteplase in acute ischaemic stroke (ECASS II). Second European-Australasian acute stroke study investigators. Lancet. (1998) 352:1245–51. 10.1016/S0140-6736(98)08020-9 9788453

[12] Wahlgren N Ahmed N Dávalos A Ford GA Grond M Hacke W. Thrombolysis with alteplase for acute ischaemic stroke in the Safe Implementation of Thrombolysis in Stroke-Monitoring Study (SITS-MOST): an observational study. Lancet. (2007) 369:275–82. 10.1016/S0140-6736(07)60149-4 17258667

[13] Hacke W Kaste M Bluhmki E Brozman M Dávalos A Guidetti D. Thrombolysis with Alteplase 3 to 4. 5 Hours after Acute Ischemic Stroke. N Engl J Med. (2008) 359:1317–29. 10.1056/NEJMoa0804656 18815396

[14] IST-3 collaborative group. The benefits and harms of intravenous thrombolysis with recombinant tissue plasminogen activator within 6 h of acute ischaemic stroke (the third international stroke trial [IST-3]): a randomised controlled trial. Lancet. (2012) 379:2352–63. 10.1016/S0140-6736(12)60768-5 22632908 PMC3386495

[15] Keselman B Gdovinová Z Jatuzis D Melo TPE Vilionskis A Cavallo R. Safety and outcomes of intravenous thrombolysis in posterior versus anterior circulation stroke: results from the safe implementation of treatments in stroke registry and meta-analysis. Stroke. (2020) 51:876–82. 10.1161/STROKEAHA.119.027071 31914885

[16] Macha K Hoelter P Siedler G Knott M Schwab S Doerfler A. Multimodal CT or MRI for IV thrombolysis in ischemic stroke with unknown time of onset. Neurology. (2020) 95:e2954–64. 10.1212/WNL.0000000000011059 33087492

[17] Kerenyi L Kardos L Szász J Szatmári S Bereczki D Hegedüs K. Factors influencing hemorrhagic transformation in ischemic stroke: a clinicopathological comparison. Eur J Neurol. (2006) 13:1251–5. 10.1111/j.1468-1331.2006.01489.x 17038041

[18] Tan S Wang D Liu M Zhang S Wu B Liu B. Frequency and predictors of spontaneous hemorrhagic transformation in ischemic stroke and its association with prognosis. J Neurol. (2014) 261:905–12. 10.1007/s00415-014-7297-8 24590407

[19] Hwang DY Silva GS Furie KL Greer DM. Comparative sensitivity of computed tomography vs. magnetic resonance imaging for detecting acute posterior fossa infarct. J Emerg Med. (2012) 42:559–65. 10.1016/j.jemermed.2011.05.101 22305149 PMC3346849

[20] Ganesh A Menon BK Assis ZA Demchuk AM Al-Ajlan FS Al-Mekhlafi MA. Discrepancy between post-treatment infarct volume and 90-day outcome in the ESCAPE randomized controlled trial. Int J Stroke. (2020) 16:593–601. 10.1177/1747493020929943 32515694

[21] Zürcher E Richoz B Faouzi M Michel P. Differences in Ischemic anterior and posterior circulation strokes: a clinico-radiological and outcome analysis. J Stroke Cerebrovas Dis. (2019) 28:710–8. 10.1016/j.jstrokecerebrovasdis.2018.11.016 30501979

[22] Sommer P Posekany A Serles W Marko M Scharer S Fertl E. Is functional outcome different in posterior and anterior circulation stroke? Stroke. (2018) 49:2728–32. 10.1161/STROKEAHA.118.021785 30355215

[23] Subramanian G Silva J Silver FL Fang J Kapral MK Oczkowski W. Risk factors for posterior compared to anterior ischemic stroke: an observational study of the registry of the Canadian stroke network. Neuroepidemiology. (2009) 33:12–6. 10.1159/000209282 19299902

[24] Linfante I Llinas RH Schlaug G Chaves C Warach S Caplan LR. Diffusion-weighted imaging and national institutes of health stroke scale in the acute phase of posterior-circulation stroke. Arch Neurol. (2001) 58:621–8. 10.1001/archneur.58.4.621 11295993

[25] Sommer P Seyfang L Posekany A Ferrari J Lang W Fertl E. Prehospital and intra-hospital time delays in posterior circulation stroke: results from the Austrian stroke unit registry. J Neurol. (2017) 264:131–8. 10.1007/s00415-016-8330-x 27822599 PMC5225195

