# Supplementary figures and images for: IV-Thrombolysis in Ischemic Stroke With Unknown Time of Onset—Safety and Outcomes in Posterior vs. Anterior Circulation Stroke
Source: Front Neurol. 2021 Aug 27;12:692067. doi: 10.3389/fneur.2021.692067 (PMC8430341; doi:10.3389/fneur.2021.692067)

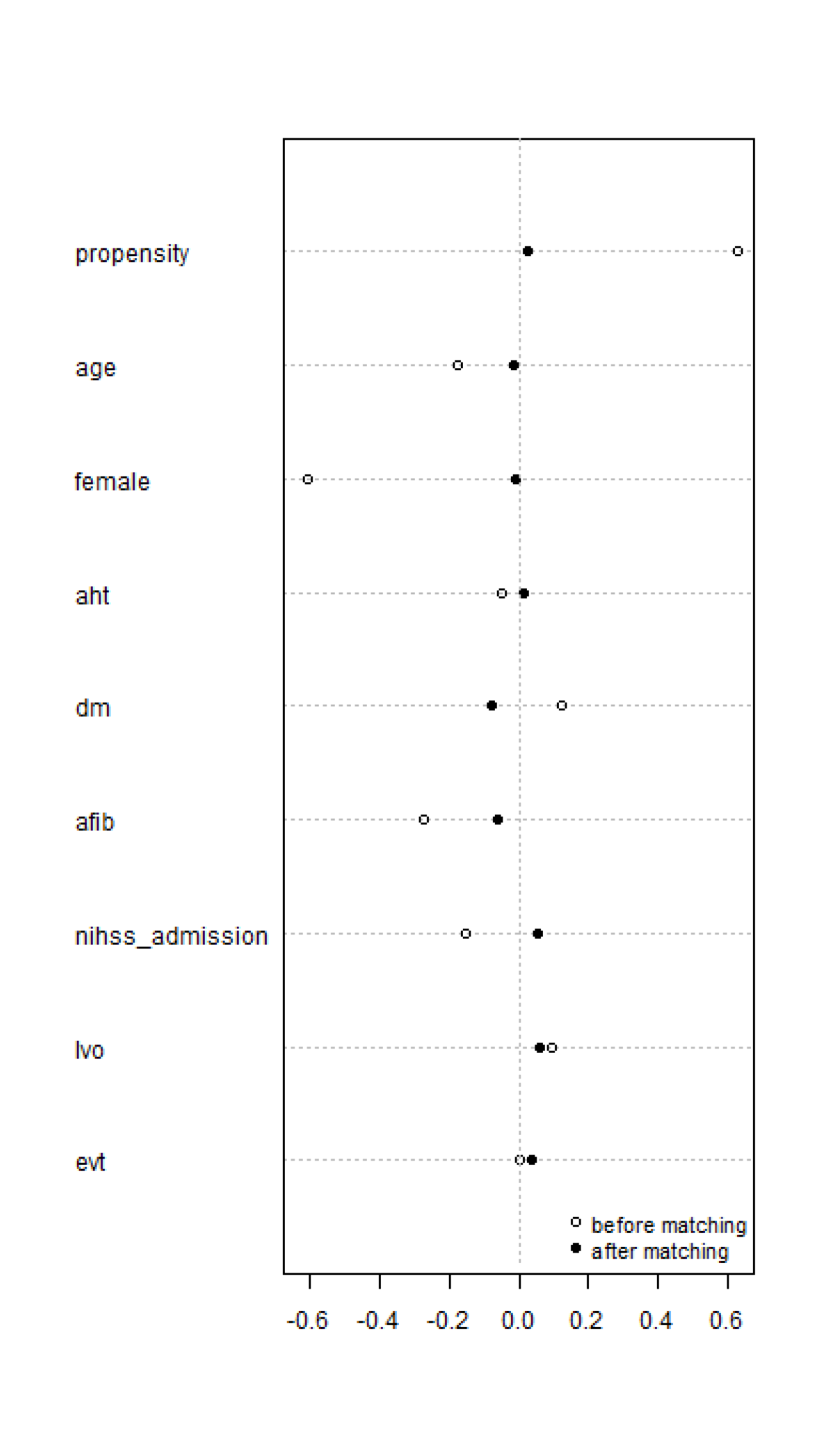

Supplement: Supplemental Figure 1 — Standardized differences in variables included in the propensity score before and after matching in the clinico-radiologic patient cohort. [file Image_1.TIF]

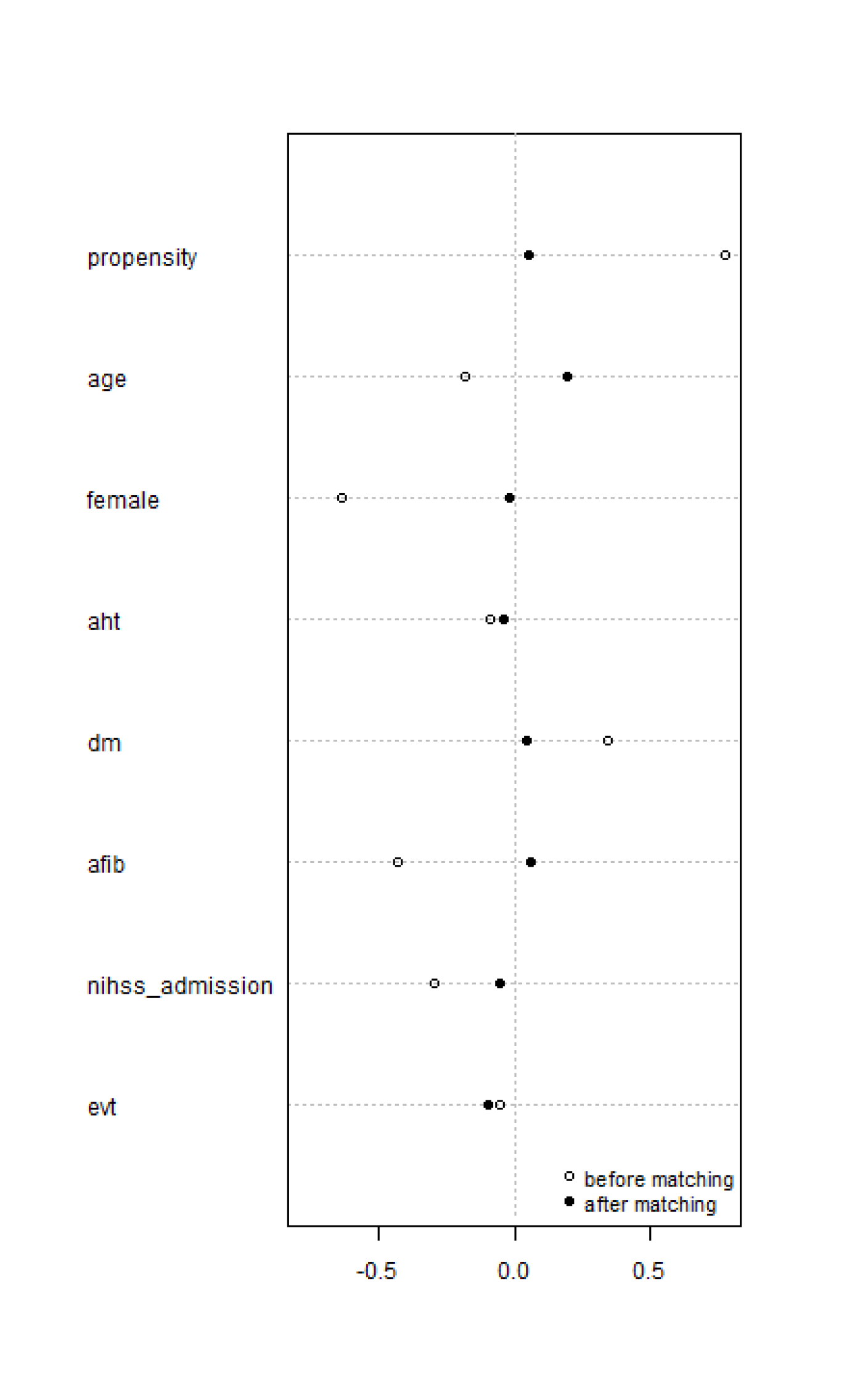

Supplement: Supplemental Figure 2 — Standardized differences in variables included in the propensity score before and after matching in the large vessel occlusion patient cohort. [file Image_2.TIF]
